# The Philosophy of the Moving Powers of the Blood

**Published:** 1845-06

**Authors:** 


*The Philosophy of the moving powers of the Blood.* By G. Calvert Holland, M. D., Physician Extraordinary to the Sheffield General Infirmary; formerly President of the Hunterian and Royal Physical Societies, Edinburgh; and Bachelor of Letters of the University of Paris. (With a motto.) 8vo. pp. 308. London, 1844.

It may appear trite to remark, that without a knowledge of the forces that propel the blood through the system it is impossible to comprehend fully any disease to which the frame is liable,—seeing that such disease, even if not evinced by marked disturbance in the general circulatory movements, must be witnessed in the system of nutrition, or in other words, in the capillary vessels;—for it is through them, that all nutrition and secretion are accomplished. Impressed with the importance of a full acquaintance with the moving powers of the blood, the author of the work before us, which he has had the kindness to transmit to us, has entered into an elaborate and intelligent inquiry into the evidences brought forward, as regards the agency of the heart, arteries, capillary vessels and veins; and although he has not adduced any very novel facts or arguments, his general conclusions appear to us to be sound, and in accordance with the best opinions. We were already familiar with them, from their having appeared in the pages of the Edinburgh Medical and Surgical Journal; and it was owing to "the most flattering commendations from some of the highest physiological authorities," that they appear in their present form—having undergone great alterations, with many additions, and a most careful revision.

